# New Insights on Structures Forming the Lignin-Like Fractions of Ancestral Plants

**DOI:** 10.3389/fpls.2021.740923

**Published:** 2021-10-07

**Authors:** Jorge Rencoret, Ana Gutiérrez, Gisela Marques, José C. del Río, Yuki Tobimatsu, Pui Ying Lam, Marta Pérez-Boada, Francisco Javier Ruiz-Dueñas, José M. Barrasa, Angel T. Martínez

**Affiliations:** ^1^Instituto de Recursos Naturales y Agrobiología de Sevilla (IRNAS), CSIC, Seville, Spain; ^2^Research Institute for Sustainable Humanosphere, Kyoto University, Kyoto, Japan; ^3^Centro de Investigaciones Biológicas “Margarita Salas” (CIB), CSIC, Madrid, Spain; ^4^Departamento de Biología Vegetal, Universidad de Alcalá, Alcalá de Henares, Spain

**Keywords:** lignin, flavonoids, apigenin, amentoflavone, naringenin, kaempferol, analytical pyrolysis, 2D NMR

## Abstract

In the present work, lignin-like fractions were isolated from several ancestral plants –including moss (*Hypnum cupressiforme* and *Polytrichum commune*), lycophyte (*Selaginella kraussiana*), horsetail (*Equisetum palustre*), fern (*Nephrolepis cordifolia* and *Pteridium aquilinum*), cycad (*Cycas revoluta*), and gnetophyte (*Ephedra fragilis*) species– and structurally characterized by pyrolysis-gas chromatography-mass spectrometry (Py-GC/MS) and two-dimensional nuclear magnetic resonance (2D-NMR) spectroscopy. Py-GC/MS yielded marker compounds characteristic of lignin units, except in the *H. cupressiforme*, *P. commune* and *E. palustre* “lignins,” where they were practically absent. Additional structural information on the other five samples was obtained from 2D-NMR experiments displaying intense correlations signals of guaiacyl (G) units in the fern and cycad lignins, along with smaller amounts of *p*-hydroxyphenyl (H) units. Interestingly, the lignins from the lycophyte *S. kraussiana* and the gnetophyte *E. fragilis* were not only composed of G- and H-lignin units but they also incorporated significant amounts of the syringyl (S) units characteristic of angiosperms, which appeared much later in plant evolution, most probably due to convergent evolution. The latter finding is also supported by the abundance of syringol derivatives after the Py-GC/MS analyses of these two samples. Regarding lignin structure, β−*O*−4′ alkyl-aryl ethers were the most abundant substructures, followed by condensed β−5′ phenylcoumarans and β−β′ resinols (and dibenzodioxocins in the fern and cycad lignins). The highest percentages of alkyl-aryl ether structures correlated with the higher S/G ratio in the *S. Kraussiana* and *E. fragilis* lignin-like fractions. More interestingly, apart from the typical monolignol-derived lignin units (H, G and S), other structures, assigned to flavonoid compounds never reported before in natural lignins (such as amentoflavone, apigenin, hypnogenol B, kaempferol, and naringenin), could also be identified in the HSQC spectra of all the lignin-like fractions analyzed. With this purpose, *in vitro* synthesized coniferyl-naringenin and coniferyl-apigenin dehydrogenation polymers were used as standards. These flavonoids were abundant in *H. cupressiforme* appearing as the only constituents of the moss lignin-like fraction (including 84% of dimeric hypnogenol B) and their abundance decreased in those of *S. Kraussiana* (with amentoflavone and naringenin representing 14% of the total aromatic units), and the two ancient gymnosperms (0.4–1.2%) and ferns (0–0.7%).

## Introduction

The first land plants to colonize Earth, which evolved from freshwater green algae (Charophyceae), appeared around 450 Mya as confirmed by the fossil record (Kenrick and Crane, 1997). Because of the switch from an aqueous to a gaseous medium, early land plants faced key challenges related to increased environmental stresses (such as temperature fluctuations, desiccation, UV radiation, scarce nutrients, etc.). Therefore, land plants required significant metabolic adaptations to produce UV shields, antioxidants, and precursors for structural biopolymers to resist desiccation, improve light interception, and successfully adapt to terrestrial environments. In more advanced terrestrial plants, the aromatic lignin polymer (Weng and Chapple, 2010; Ralph et al., 2019) assumed some of the aforementioned functions (Raven, 1984; Qian et al., 2015; Xie et al., 2018). However, before land plants developed the ability to synthesize lignin, it is believed that flavonoid compounds were responsible for protecting them from UV radiation (Clayton et al., 2018; Davies et al., 2020).

Flavonoids are a large family of secondary metabolites ubiquitously found in land plants (Grotewold, 2006). These phenolic compounds, with a C6-C3-C6 carbon framework, are synthesized from phenylalanine and malonyl-CoA through the phenylpropanoid pathway. Flavonoids are usually found in either free and glycosylated forms. However, and especially thanks to the great advances in two-dimensional nuclear magnetic resonance (2D-NMR) spectroscopy during the last two decades, the presence of flavonoids incorporated into the lignin polymer has been discovered, acting as natural lignin monomers together with other phenolic precursors biosynthesized outside the monolignol pathway (del Río et al., 2020, 2021). In fact, all flavonoids with a free 4′-OH in the B-ring are potentially susceptible of being enzymatically oxidized, forming radicals compatible with lignification in cross-coupling reactions with monolignols.

The flavone tricin was the first discovered lignin-incorporated flavonoid, identified in wheat straw (del Río et al., 2012). Later, tricin was also found in many others monocots, especially from the Poaceae family (Rencoret et al., 2013, 2015; Lan et al., 2016b). The way tricin is incorporated into the lignin and its compatibility in cross-coupling reactions with monolignols have been studied extensively during the last years (Lan et al., 2015, 2016a). Furthermore, studies with genetically modified plants have demonstrated that some flavonoid intermediates in the tricin biosynthetic pathway, such as apigenin and naringenin, can also be incorporated into the lignin polymers of mutant plants (Lam et al., 2017, 2019), providing further evidence on the ability of flavonoids to act as true lignin monomers participating in cross-coupling reaction with traditional monolignols (del Río et al., 2020, 2021). Whereas in primitive plants, both free and lignin-incorporated flavonoids could contribute to plant protection against UV radiation, flavonoids in the lignin of more evolved angiosperms, such as grasses, would have a structural function as initiation points of the lignin chains, and their removal results in transgenic plants with lower lignin content as shown by blocking the biosynthesis of tricin (Lam et al., 2017).

**FIGURE 1:**
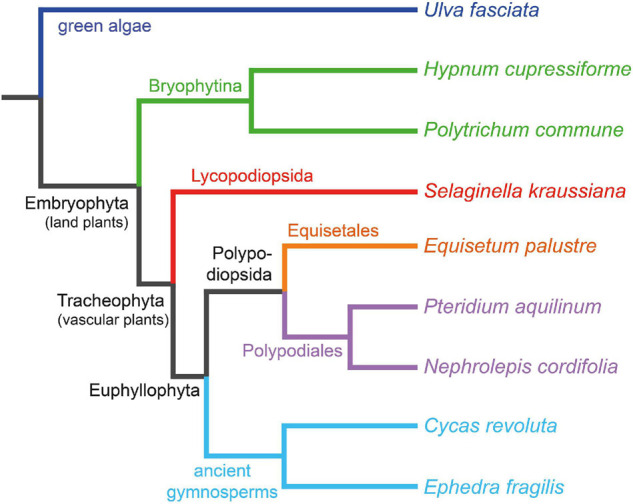
Phylogenetic tree of the eight ancestral plants whose lignin-like fractions are analyzed, with the green alga *Ulva fasciata* Delile as an outsider to root the tree. Generated by phyloT v2 (https://phylot.biobyte.de/about.cgi).

The present work aims to study how lignin has evolved among different ancestral plants – from one of the most ancestral groups of terrestrial plants (mosses) to more evolved primitive gymnosperms − and to determine if flavonoids are present in their lignin-like fractions. For this purpose, lignin-like fractions were isolated by the dioxane method (Rencoret et al., 2015) from eight ancestral plants whose phylogenetic relationships are shown in Figure 1—including mosses *Hypnum cupressiforme* Hedw. and *Polytrichum commune* Hedw., lycophyte *Selaginella kraussiana* (Kunze) A. Braun, horsetail *Equisetum palustre* L., ferns *Nephrolepis cordifolia* (L.) C. Presl and *Pteridium aquilinum* (L.) Kuhn, cycad *Cycas revoluta* Thunb., and gnetophyte *Ephedra fragilis* Desf.– and their composition and structure were thoroughly characterized by a combination of analytical pyrolysis and 2D NMR.

## Materials and Methods

### Ancestral Plant Materials

The plant material used in this study was collected from different locations: (i) *P. commune* and *E. palustre* from Rascafría (Madrid, Spain); (ii) *H. cupressiforme* and *P. aquilinum* from Burguete (Navarre, Spain); and (iii) *C. revoluta*, *S. kraussiana*, *N. cordifolia* and *E. fragilis* from the Juan Carlos I Royal Botanic Gardens (University of Alcalá, Madrid). The samples were air-dried and milled using a knife mill (Janke and Kunkel, Analysemühle). Non-structural lipophilic and hydrophilic compounds were removed by successive extractions with acetone, 80% ethanol, and water prior the isolation of lignin-like material. The extraction was carried out with acetone in a Soxhlet apparatus for 8 h, and then with 80% ethanol (3 × 30 min) and water (3 × 30 min) in an ultrasonic bath.

### Isolation of Lignin-Like Fractions From Ancestral Plants

Lignin-like fractions were isolated by the dioxane method with minor modifications (Evtuguin et al., 2001; Rencoret et al., 2015). Briefly, 8 g of the pre-extracted plant samples were refluxed under N_2_ (3 × 40 min, using fresh solution each time) with 80 mL of 0.2 M HCl in dioxane-water (9:1, v/v). A final extraction was performed without addition of HCl in the dioxane-water mixture. The liquid phases were filtered and concentrated separately in a rotary evaporator at 40°C (up to approximately 20 mL) to prevent lignin precipitation. Then, the concentrates were combined and precipitated in cold-water (1,500 mL) under stirring, centrifuged, and freeze dried. Finally, they were extracted with 200 mL of diethyl ether to remove low molecular weight contaminants. The dioxane lignin-like fractions yield represented 1–5% of the plant material.

### Gel Permeation Chromatography

Gel permeation chromatography (GPC) analysis of isolated lignin-like fractions was carried out on a Prominence-i LC-2030 3D GPC system (Shimadzu, Kyoto, Japan) equipped with a photodiode array detector and a PLgel MIXED-D column (Agilent Technologies, Stockport, United Kingdom), using the experimental conditions previously described (Rencoret et al., 2020).

### Pyrolysis Coupled to Gas Chromatography and Mass Spectrometry

Analytical pyrolyses of the lignin preparations (∼1 mg) were performed at 500°C (1 min) in an EGA/PY-3030D microfurnance pyrolyzer (Frontier Laboratories Ltd., Fukushima, Japan) connected to a GC equipment 7820A (Agilent Technologies, Inc., Santa Clara, CA, United States) equipped with a DB-1701 fused-silica capillary column (30 m × 0.25 mm i.d., 0.25 μm film thickness) and an Agilent 5975 MS selective detector (EI at 70 EV). The oven temperature was programmed from 50 to 100°C at 20°C min^–1^ and then ramped to 280°C at a heating rate of 6°C min^–1^, and held for 5 min. The released compounds were identified by comparison of their mass spectra with those in the literature (Ralph and Hatfield, 1991). Molar peak areas were calculated for the released lignin-derived products (specific markers) and the data for two replicates were averaged and expressed as percentages.

### 2D-NMR Spectroscopy

For NMR analyses, ∼30 mg of the lignin-like fractions were transferred into an NMR tube and dissolved in 0.6 mL of deuterated dimethylsulfoxide (DMSO-*d*_6_). Heteronuclear single quantum coherence (HSQC) spectra were acquired at 300 K on a Bruker AVANCE III 500 MHz spectrometer equipped with a 5 mm TCI cryogenic probe. The HSQC experiment was performed using a standard adiabatic pulse sequence (hsqcetgpsisp.2) and parameters already described (Rencoret et al., 2020). HSQC cross-peaks from flavonoid units were assigned by literature comparison (Lam et al., 2017, 2019), as well as by using flavonoid standards commercially available (for naringenin, kaempferol, apigenin and amentoflavone) and flavonoid-containing synthetic lignin (see below).

A semiquantitative analysis, based on HSQC signals integration, was performed using Bruker’s TopSpin software. In the aromatic/unsaturated region, the S_2,6_, G_2_, Ap_8_, K_8_ and N_6,8_ correlation signals were used to determine the relative abundances of the phenylpropane S and G, and flavonoid apigenin (Ap), kaempferol (K) and naringenin (N) units, respectively. Since the S_2,6_ and N_6,8_ signals involve two proton-carbon pairs, their integration values were divided in half. In the case of the biflavonoid structures, their relative abundances were determined by integrating the Am_6__′__′_ (amentoflavone) and Hy_2_ (hypnogenol B) signals. The C_8_/H_8_ and C_2_/H_2_ correlation signals of cinnamaldehyde end-units (J) and ferulates (FA) were used to estimate their relative abundances. For quantification of inter-unit linkages resulting in different lignin substructures, the side-chain C_α_/H_α_ cross-signals of β–*O*–4′ alkyl aryl ethers (A_α_), β–5′ phenylcoumarans (B_α_), β–β′ resinols (C_α_) and 5–5′ dibenzodioxocins (D_α_) were used.

### Standard Flavonoids and Synthetic Lignin Polymers

Amentoflavone was purchased from Selleck (Houston, TX, United States), whereas naringenin [(2S)-4′,5,7-trihydroxyflavan-4-one], kaempferol (3,4′,5,7-tetrahydroxyflavone), and apigenin (4′,5,7-trihydroxyflavone) were acquired from Sigma-Aldrich (Lyon, France). Synthetic lignins (dehydrogenation polymers, DHPs) from coniferyl alcohol/naringenin (GN-DHP) and coniferyl alcohol/apigenin (GA-DHP) were synthesized by the peroxidase-catalyzed dehydrogenative copolymerization of coniferyl alcohol (0.425 mmol) with naringenin (0.075 mmol) or apigenin (0.075 mmol), respectively, using the so-called bulk polymerization method (Tobimatsu et al., 2008) as previously described (Lam et al., 2017, 2019).

## Results and Discussion

### Molecular Weight Distributions of Lignin-Like Fractions From Ancestral Plants

The weight-average (M*_*w*_*) and number-average (M*_*n*_*) molecular weights (g mol^–1^), and polydispersity index (M*_*w*_*/M*_*n*_*) of the lignin-like fractions isolated from ancestral plants were determined by GPC analyses and are shown in Table 1. The data revealed that these fractions contain polymeric structures, as can be deduced from their M*_*w*_* values that ranged from 4,840 to 13,150 g mol^–1^. Most of the lignin-like fractions analyzed presented a M*_*w*_* comparable to other isolated lignins (Rencoret et al., 2020), with the exception of the lignin-fractions from mosses, whose M*_*w*_* were relatively higher (13,150 and 10,120 g mol^–1^ for *H. cupressiforme* and *P. commune*, respectively). Also, the polydispersity (M*_*w*_*/M*_*n*_*) values of the lignin-like fractions were slightly higher in comparison to those observed for other isolated lignins (Rencoret et al., 2020).

**TABLE 1:** Weight-average (M*_*w*_*) and number-average (M*_*n*_*) molecular weights (g mol^–1^), and polydispersity (M*_*w*_*/M*_*n*_*) of the lignin-like fractions isolated from ancestral plants.

	Plant species^*a*^							
	
	HC	PC	SK	EP	NC	PA	CR	EF
*M*_*w*_	13,150	10,120	6,630	4,840	7,290	6,220	5,640	6,800
*M*_*n*_	3,530	3,440	1,760	2,270	2,340	2,280	2,450	3,010
*M*_*w*_/*M*_*n*_	3.7	2.9	3.8	2.1	3.1	2.7	2.3	2.3

*^*a*^HC, *H. cupressiforme*; PC, *P. commune*; SK, *S. kraussiana*; EP, *E. palustre*; NC, *N. cordifolia*; PA, *P. aquilinum*; CR, *C. revoluta*; EF, *E. fragilis*.*

### Lignin Composition of Ancestral Plants as Shown by Analytical Pyrolysis

**FIGURE 2:**
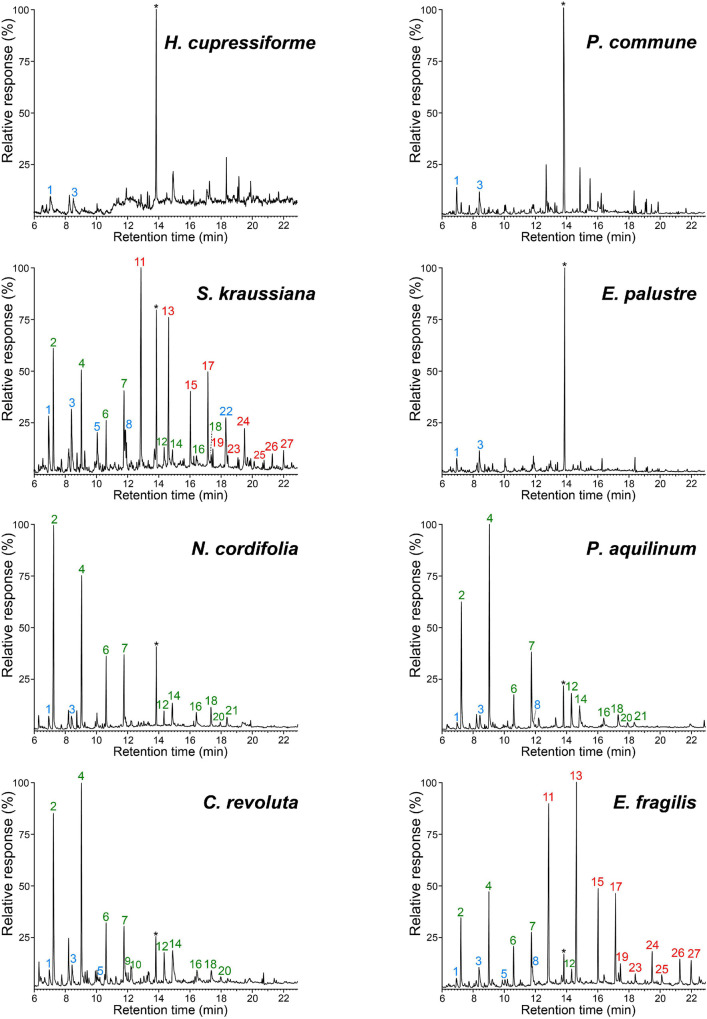
Pyrograms of the lignin-like fractions isolated from the eight ancestral plants studied. Phenolic markers of H, G, and S lignin units are labeled in blue, green and red, respectively. Peaks identities: phenol (1), guaiacol (2), 4-methylphenol (3), 4-methylguaiacol (4), 4-ethylphenol (5), 4-ethylguaicol (6), 4-vinylguaicol (7), 4-vinylphenol (8), eugenol (9), 4-propylguaiacol (10), syringol (11), *trans-*isoeugenol (12), 4-methylsyringol (13), vanillin (14), 4-ethylsyringol (15), acetovanillone (16), 4-vinylsyringol (17), guaiacylacetone (18), 4-allylsyringol (19), propiovanillone (20), guaiacyl vinyl ketone (21), 4-hydroxyacetophenone (22), *cis*-4-propenylsyringol (23), *trans*-4-propenylsyringol (24), syringaldehyde (25), acetosyringone (26), and syringylacetone (27). *Solvent stabilizer 4-methyl-2,6-ditertbutylphenol (rt ∼13.8 min).

The ancestral lignin-like fractions of the plants studied were firstly analyzed by pyrolysis coupled to gas chromatography and mass spectrometry (Py-GC/MS), a highly sensitive method that can provide information on their relative composition in terms of syringyl (S), guaiacyl (G) and *p-*hydroxyphenyl (H) units (as H:G:S ratio). By Py-GC/MS (Figure 2) all the lignin-like fractions, with the exception of those from the mosses (*H. cupressiforme*, *P. commune*) and the horsetail (*E. palustre*), released characteristic lignin markers. Despite small amounts of phenol and 4-methylphenol were found among the pyrolysis products of these three lignins, the absence of H-type markers with longer side-chains (such as 4-ethylphenol, eugenol or 4-propenylphenol) indicates that they do not actually come from lignin but more likely from other compounds, such as proteins or flavonoids that also yield these H-type compounds upon pyrolysis. It is therefore possible to conclude the absence of recognizable lignin building blocks in the mosses and horsetail studied here.

*S*-lignin markers were the most abundant in the pyrograms of the gnetophyte (*E. fragilis*) and the lycophyte (*S. kraussiana*) lignin-like fractions, while G-lignin markers predominated in the rest of samples, as in the cycad (*C. revoluta*), and the ferns (*P. aquilinum* and *N. cordifolia*) lignins. The pyrogram of the *S. kraussiana* fraction showed an unusually high peak of 4-hydroxyacetophenone (peak 22), which was absent in other H-rich lignins (Rencoret et al., 2016). Therefore, it would be logical to think that it is not derived from H-lignin but comes from other lignin-related compounds containing *p*-hydroxyphenyl moieties in their structures, as will be shown below.

### In-Depth Structural Analysis of Ancestral Lignins by 2D-NMR

**FIGURE 3:**
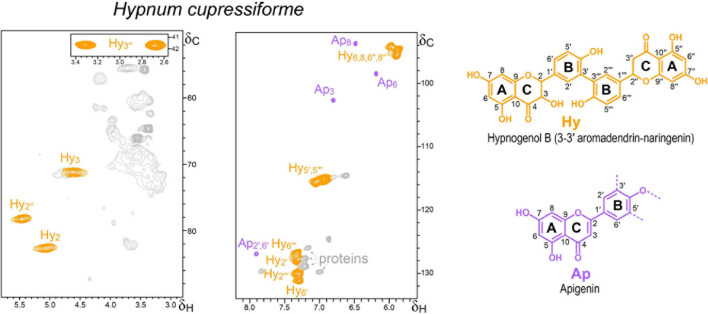
Aliphatic-oxygenated (δ_*C*_/δ_*H*_ 50–90/2.8–5.8) and aromatic (δ_*C*_/δ_*H*_ 92–134/5.6–8.2) regions of the HSQC spectrum of the lignin-like preparation isolated from the moss *H. cupressiforme*. The main signals correspond to the flavonoids apigenin (Ap) and hypnogenol B (Hy), whose structures are shown on the right.

**FIGURE 4:**
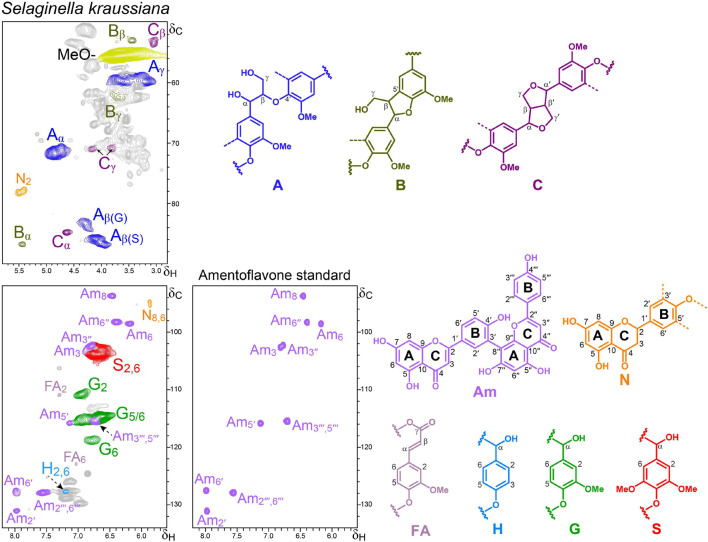
Aliphatic-oxygenated (δ_*C*_/δ_*H*_ 50–90/2.8–5.8) and aromatic (δ_*C*_/δ_*H*_ 92–134/5.5–8.2) regions of the HSQC spectrum of the *S. kraussiana* lignin-like preparation. The HSQC spectrum of authentic amentoflavone is also shown for comparison. The main structures identified – corresponding to β–*O*–4′ ether (A), β–5′ phenylcoumaran (B) and β–β′ resinols (C) side chains, *p*-hydroxyphenyl (H), guaiacyl (G) and syringyl (S) units, and ferulic acid (FA), amentoflavone (Am) and naringenin (N) moieties – are depicted.

**FIGURE 5:**
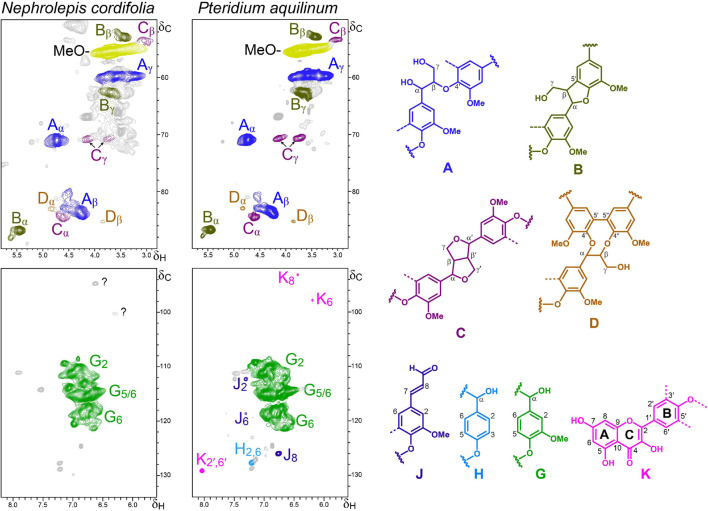
Aliphatic-oxygenated (δ_*C*_/δ_*H*_ 50–90/2.8–5.8) and aromatic (δ_*C*_/δ_*H*_ 92–134/5.5–8.0) regions of the HSQC spectra of lignin-like preparations isolated from the ferns *N. cordifolia* and *P. aquilinum*. The main structures identified – corresponding to β–*O*–4′ ether (A), β–5′ phenylcoumaran (B), β–β′ resinols (C) and 5–5′ dibenzodioxocins (D) side chains, *p*-hydroxyphenyl (H) and guaiacyl (G) units, coniferaldehyde end-groups (J), and kaempferol moieties (K) – are shown on the right.

**FIGURE 6:**
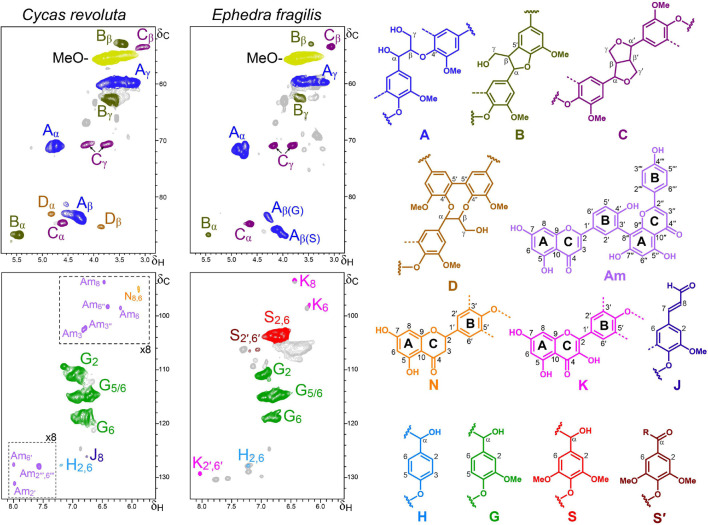
Aliphatic-oxygenated (δ_*C*_/δ_*H*_ 50–90/2.8–5.8) and aromatic (δ_*C*_/δ_*H*_ 92–134/5.5–8.2) regions of the HSQC spectra of the *C. revoluta* and *E. fragilis* lignin-like preparations. The main structures identified – corresponding to β–*O*–4′ ether (A), β–5′ (B), β–β′ (C) and 5–5′ (D) side chains, *p*-coumaryl (H), guaiacyl (G) and syringyl (S and S′) units, coniferaldehyde end-groups (J), and naringenin (N), kaempferol (K) and amentoflavone (Am) moieties – are depicted on the right. The dash-line boxes in the *C. revoluta* spectrum are scaled-up (×8 intensity).

After the initial Py-GC/MS study of lignin composition, 2D-NMR analyses of the isolated lignin-like fractions provided additional valuable information about their composition in flavonoid and conventional lignin (H, G, and S) units, as well as on their inter-unit linkages resulting in different substructures. The main correlation signals identified in the HSQC spectra discussed in the following subsections (Figures 3–6) are listed in Table 2, and a semiquantitative summary of the different spectra is provided in Table 3. Significant differences were found in the composition and structure of the lignin-like preparations from the different ancestral plants, as detailed below.

**TABLE 2:** Assignments of the ^1^H/^13^C signals identified in the HSQC spectra (Figures 3–6) of the lignin-like fractions isolated from the ancestral plants studied.

Label	δ_*C*_/δ_*H*_	Assignment
N_3_/Hy_3__′__′_	41.8/2.69, 3.31	C_3_/H_3_ in naringenin (N)/C_3__′__′_/H_3__′__′_ in hypnogenol B (Hy)
B_β_	52.9/3.43	C_β_/H_β_ in phenylcoumarans (B)
C_β_	53.3/3.05	C_β_/H_β_ in β–β′resinols (C)
MeO	55.5/3.71	C/H in aromatic methoxy group
A_γ_	59.7/3.21, 3.59	C_γ_/H_γ_ in β–*O*–4′ alkyl-aryl ethers (A)
B_γ_	62.6/3.68	C_γ_/H_γ_ in phenylcoumarans (B)
C_γ_	70.7/3.73, 4.14	C_γ_/H_γ_ in β–β′resinols (B)
A_αG_	70.9/4.72	C_α_/H_α_ in β–*O*–4′ alkyl-aryl ethers (A) linked to a G unit
Hy_3_	71.2/4.62	C_3_/H_3_ in hypnogenol B (Hy)
A_αS_	71.7/4.84	C_α_/H_α_ in β–*O*–4′ alkyl-aryl ethers (A) linked to a S unit
N_2_	78.1/5.49	C_2_/H_2_ in naringenin (N)
Hy_2__′__′__′_	78.3/5.46	C_2__′__′_/H_2__′__′_ in hypnogenol B (Hy)
Hy_2_	82.7/5.07	C_2_/H_2_ in hypnogenol B (Hy)
D_α_	83.0/4.82	C_α_/H_α_ in 5–5′ dibenzodioxocins (D)
A_β__G_	83.7/4.27	C_β_/H_β_ in β–*O*–4′ alkyl-aryl ethers (A) linked to a G unit
C_α_	84.8/4.62	C_α_/H_α_ in β–β′resinols (C)
D_β_	85.3/3.85	C_β_/H_β_ in 5–5′ dibenzodioxocins (D)
A_β__S_	86.4/4.03	C_β_/H_β_ in β–*O*–4′ alkyl-aryl ethers (A) linked to a S unit
B_α_	86.7/5.45	C_α_/H_α_ in phenylcoumarans (B)
K_8_	93.3/6.43	C_8_/H_8_ in kaempferol (K)
Ap_8_	93.7/6.46	C_8_/H_8_ in apigenin (Ap)
Am_8_	93.8/6.46	C_8_/H_8_ in amentoflavone (Am)
N_8_/Hy_8_/Hy_8__′__′__′_	94.6/5.87	C_8_/H_8_ in naringenin (N)/C_8_/H_8_ and C_8__′__′_/H_8__′__′_ in hypnogenol B (Hy)
N_6_/Hy_6_/Hy_6__′__′_	95.5/5.87	C_6_/H_6_ in naringenin (N)/C_6_/H_6_ and C_6__′__′_/H_6__′__′_ in hypnogenol B (Hy)
K_6_	97.9/6.20	C_6_/H_6_ in kaempferol units (K)
Am_6__′__′_	98.3/6.40	C_6__′__′_/H_6__′__′_ in amentoflavone (Am)
Ap_6_	98.5/6.18	C_6_/H_6_ in apigenin (Ap)
Am_6_	98.5/6.18	C_6_/H_6_ in amentoflavone (Am)
Am_3__′__′_	102.2/6.77	C_3__′__′_/H_3__′__′_ in amentoflavone (Am)
Ap_3_	102.7/6.79	C_3_/H_3_ in apigenin units (Ap)
Am_3_	102.7/6.82	C_3_/H_3_ in amentoflavone (Am)
S_2,6_	103.9/6.68	C_2_/H_2_ and C_6_/H_6_ in syringyl units (S)
S′_2,6_	106.1/7.30	C_2_/H_2_ and C_6_/H_6_ in Cα-oxidized syringyl units (S′)
G_2_	110.8/6.96	C_2_/H_2_ in guaiacyl units (G)
FA_2_	110.9/7.29	C_2_/H_2_ in ferulates (FA)
J_2_	112.4/7.31	C_2_/H_2_ in coniferaldehyde end-groups (J)
G_5/6_	114.9/6.80	C_5_/H_5_ and C_6_/H_6_ in guaiacyl units (G)
K_3__′__,5__′_	115.1/6.91	C_3__′_/H_3__′_ and C_5__′_/H_5__′_ in kaempferol (K)
N_3__′__,5__′_	115.2/6.92	C_3__′_/H_3__′_ and C_5__′_/H_5__′_ in naringenin (N)
Am_3__′__′__′__,5__′__′__′_	115.3/6.72	C_3__′__′__′_/H_3__′__′__′_ and C_5__′__′__′_/H_5__′__′__′_ in amentoflavone (Am)
Ap_3__′__,5__′_	115.5/6.94	C_3__′_/H_3__′_ and C_5__′_/H_5__′_ in apigenin (Ap)
Am_5__′_	115.8/7.14	C_5__′_/H_5__′_ in amentoflavone (Am)
J_6_	118.7/7.30	C_6_/H_6_ in coniferaldehyde end-groups (J)
G_6_	118.9/6.79	C_6_/H_6_ in guaiacyl units (G)
FA_6_	123.2/7.11	C_6_/H_6_ in ferulates (FA)
J_8_	126.0/6.76	C_8_/H_8_ in coniferaldehyde end-groups (J)
Am_6__′_	127.5/7.99	C_6__′_/H_6__′_ in amentoflavone (Am)
H_2,6_	127.6/7.19	C_2_/H_2_ and C_6_/H_6_ in *p*-coumaryl units (H)
N_2__′__,6__′_	127.8/7.29	C_2__′_/H_2__′_ and C_6__′_/H_6__′_ in naringenin (N)
Am_2__′__′__′__,6__′__′__′_	127.9/7.56	C_2__′__′__′_/H_2__′__′__′_ and C_6__′__′__′_/H_6__′__′__′_ in amentoflavone (Am)
K_2__′__,6__′_	129.2/8.02	C_2__′_/H_2__′_ and C_6__′_/H_6__′_ in kaempferol (K)
Am_2__′_	131.0/7.98	C_2__′_/H_2__′_ in amentoflavone (Am)
J_7_	153.5/7.62	C_7_/H_7_ in coniferaldehyde end-groups (J)

**TABLE 3:** Semiquantitative analysis of lignin aromatic units (flavonoid units included) and different substructures, from integration of HSQC spectra (see Figures 3–6 also showing structural formulae) of the lignin-like fractions from ancestral plants.

	Plant species^*a*^					
	
	HC	SK	NC	PA	CR	EF
**Aromatic units (%)^*b*^:**						
*p*-Hydroxyphenyl (H)	−	5.8	−	0.5	0.7	0.5
Guaiacyl (G)	−	25.9	100	98.8	98.7	27.0
Syringyl (S)	−	54.2	−	−	−	71.3
Naringenin (N)	−	2.5	−	−	0.2	−
Apigenin (Ap)	16.0	−	−		−	−
Kaempferol (K)	−	−	−	0.7	−	1.2
Amentoflavone (Am)	−	11.6	−	−	0.4	
Hypnogenol B (Hy)	84.0	−	−	−	−	−
**Lignin substructures (%)^*c*^_:_**						
β–*O*–4′ Alkyl-aryl ethers (A)	−	88.9 (57.5)	66.2 (19.6)	58.3 (28.6)	59.5 (32.6)	81.2 (54.6)
β–5′ Phenylcoumarans (B)	−	6.4 (4.2)	25.3 (7.5)	26.8 (13.1)	24.9 (13.6)	7.0 (4.7)
β–β′ Resinols (C)	−	4.7 (3.0)	5.5 (1.6)	5.5 (2.7)	5.4 (3.0)	10.6 (7.1)
5–5′ Dibenzodioxocins (D)	−	−	3.0 (0.9)	3.9 (1.9)	5.9 (3.2)	−
Coniferaldehyde end-groups (J)	−	−	−	5.5 (2.7)	4.3 (2.3)	1.2 (0.8)

*^*a*^HC, *H. cupressiforme*; SK, *S. kraussiana*; NC, *N. cordifolia*; PA, *P. aquilinum*; CR, *C. revoluta*; EF, *E. fragilis*.*

*^*b*^As percentage of total aromatic units (H + G + S + N + Ap + K + Am + Hy = 100).*

*^*c*^As percentage of total substructures (A + B + C + D + J = 100), with values in parentheses referred to classical lignin units (H + G + S = 100).*

*−, not detected.*

#### Mosses

Several studies indicate that liverworts and mosses were the earliest colonizers of the land (Kenrick and Crane, 1997). The presence of significant amounts of the classical lignin structures (typically formed by H, G and/or S units) in the mosses *H. cupressiforme* (class Bryopsida) and *P. commune* (class Polytrichopsida) could be discarded by the Py-GC/MS analyses as discussed above. However, the presence of flavonoid-type polyphenols was suggested by the HSQC analyses, as discussed below.

The HSQC spectrum of the lignin-like fraction from *H. cupressiforme* (Figure 3) displayed correlation signals of flavonoids and proteins. The chemical shifts of the strongest flavonoid signals were consistent with hypnogenol B, a biflavonoid composed of aromadendrin [(2R,3R)-3,4′,5,7-tetrahydroxyflavan-4-one] and naringenin linked by a 3-3′ bond, which has been found in the extracts of *H. cupressiforme* (Sievers et al., 1992). The characteristic signals of the naringenin C-ring were clearly observed at 78.3/5.46 (Hy_2″_) and 41.8/2.69 and 41.8/3.3 (Hy_3″_) ppm, whereas the equivalent signals of the aromadendrin C-ring seem to be those detected at 82.7/5.07 (Hy_2_) and 71.2/4.62 ppm (Hy_3_) (these assignments could not be fully confirmed since hypnogenol B is not commercially available). In addition to hypnogenol B, distinctive HSQC signals of the A- and C-rings of the flavone apigenin were also detected. These and other signals were assigned using as reference the HSQC spectra of authentic flavonoid standards as well as DHPs prepared by copolymerization of apigenin or naringenin with coniferyl alcohol (Supplementary Figure 1). Ap_8_, Ap_6_, and Ap_3_ signals matched well with those of the apigenin authentic standard. In contrast, the signals of apigenin B-ring did not exactly match those of free apigenin (this is only appreciable for the Ap_2__′__,6__′_ since Ap_3__′__,5__′_ would overlap with the hypnogenol B Hy_5__′__,5__′__′__′_ signal), indicating that apigenin in the *H. cupressiforme* sample could be linked through its B-ring.

On the contrary, the HSQC spectrum of the *P. commune* lignin-like fraction preparation was saturated by protein signals (Supplementary Figure 2). Although trace amounts of G- and H-lignin units had been reported in *P. commune* after cupric oxide alkaline degradation of whole material (Logan and Thomas, 1985), we could only detect H-type signals upon pyrolysis, which could arise from protein moieties. The HSQC spectrum of *P. commune* confirmed the occurrence of proteins (Kim et al., 2017) and the absence of H-lignin units in the lignin-like preparation isolated from this moss. The presence of lignin in mosses is controversial since lignin was always thought to be a unique component of vascular plants (Erickson and Miksche, 1974). Moreover, as in the case of the *H. cupressiforme* lignin-like preparation, some flavonoid signals (tentatively assigned to naringenin and kaempferol) could be also present in the *P. commune* “lignin,” albeit with low intensities and overlapped with the protein signals.

Interestingly, the flavonoids tentatively identified in the moss “lignin-like” preparation (as well as in all the other lignin-like fractions) present a B-ring structurally similar to the H-lignin units. Perhaps, this is the reason why a previous study that intended to clarify the presence/absence of lignin in *Sphagnum magellanicum* Brid. by ^13^C NMR reported the presence of H-lignin in this moss (Nimz and Tutschek, 1977). However, the characteristic signals of lignin linkages were lacking and the most probable conclusion is that the signals detected by the authors corresponded to flavonoids with an H-type B-ring and not to real H-lignin units.

#### Lycophytes

Lycophytes (class Lycopodiopsida) appeared about 400 Mya and are among the earliest terrestrial vascular plants, with the Selaginellaceae family including more than 700 species (Jermy, 1990). Their characteristic giant species, represented by extinct arborescent plants with secondary thickening in their trunks and rhizomorphs (such as *Lepidodendron* and *Sigillaria* in the order Lepidodendrales, and *Pleuromeia* in the order Isoetales), dominated the Earth flora during the Carboniferous period. Selaginellaceae together with two other lycophyte families (Lycopodiaceae and Isoetaceae) constitute the oldest lineage of vascular plants surviving on Earth (Banks, 2009). Interestingly, reduction of vegetative structures was a recurrent phenomenon along lycophyte evolution (Bateman, 1996). Thus, extant *Selaginella* and *Isoetes* are herbaceous and rhizomatous species maintaining reminiscences of giant arborescent lineages with secondary thickening. Among the *Selaginella* species, *S. kraussiana* was selected for this study since its lignin has not been studied before.

The HSQC analysis of the lignin-like preparation obtained from *S. kraussiana* (Figure 4) reveals that this primitive vascular plant has a lignin composed of not only G- and H-units, but also of significant amounts of S-units. The latter are characteristic of angiosperms that appeared much later in time, which indicates convergent evolution of S-lignin in lycophytes and angiosperms. The presence of S units in the lignin of other *Selaginella* species has been previously shown by different techniques, including CuO alkaline degradation (Logan and Thomas, 1985), acidolysis, nitrobenzene oxidation, FTIR, ozonation, ^1^H-NMR (Jin et al., 2005, 2007a), derivatization followed by reductive cleavage (Weng et al., 2008), and 2D-NMR (Weng et al., 2011). The aliphatic oxygenated region of the HSQC spectrum shows the methoxyl signal together with those of typical lignin substructures (Figure 4). β−*O*−4′ alkyl-aryl ethers (A) are the most abundant linkages (89% of the total) observed in the HSQC spectrum of the *S. kraussiana* lignin-like fraction, followed by lower amounts (around 5%) of β−5′ phenylcoumarans (B) and β−β′ resinols (C) (Table 3).

Together with the aforementioned lignin signals, the aromatic region of the HSQC spectrum of the *S. kraussiana* lignin-like fraction also showed signals of flavonoids (Figure 4). At a first glance, several of them seemed to match well with those of the flavone apigenin (Supplementary Figure 1) but a more extensive analysis revealed that they actually corresponded to the biflavonoid amentoflavone (bis-apigenin coupled at 8 and 3′ positions) and the flavanone naringenin, the latter being less abundant. The identities of these signals were confirmed by spectral comparison with authentic flavonoid standards and synthetic DHPs (Supplementary Figure 1).

Among over 130 natural products reported from *Selaginella* species (Weng and Noel, 2013), amentoflavone is the most common (bi)flavonoid (Setyawan, 2019), being also present in Euphorbiaceae, Calophyllaceae and other plants, and exhibiting a variety of pharmacological properties (Yu et al., 2017). Free amentoflavone can be easily isolated from *Selaginella* plants by ethanol extraction (Swamy et al., 2006). So, considering that the *S. kraussiana* material analyzed was successively and exhaustively extracted with several solvents (acetone, ethanol, and water), and the dioxane-lignin preparation was purified as well (with diethyl ether), it is logical to think that the amentoflavone present in this fraction is somehow bound to the lignin polymer. Another fact supporting this hypothesis is the relatively lower intensities of the Am_5__′_ and Am_2__′_ signals (compared to Am_6_, Am_8_, Am_6__′__′_ and Am_2__′__′__′__,6__′__′__′_), which suggests that amentoflavone would be connected to the lignin through its B-ring. It is believed that biflavonoids are biosynthesized through dimerization of monomeric flavonoids *via* radical coupling reactions catalyzed by peroxidases (and/or laccases) (Yamaguchi and Kato, 2012) in the same way in which lignin polymerizes from monolignols (Ralph et al., 2004).

A “milled-wood lignin” (MWL) preparation was also isolated from *S. kraussiana* by the classical procedure (Björkman, 1956), which is expected to be representative of the native lignin (Fujimoto et al., 2005). The HSQC spectrum of the *S. kraussiana* MWL was very similar to that of the dioxane-lignin (Supplementary Figure 3) and also displayed amentoflavone signals with almost identical relative abundances, reinforcing the idea that this biflavonoid might be incorporated into the lignin structure. More research is currently in progress to confirm whether the amentoflavone is covalently linked to the lignin in this *Selaginella* species.

Interestingly, in a previous work, signals for amentoflavone can also be clearly observed in the HSQC spectrum of the lignin isolated from *Selaginella moellendorffii* Hieron., although they were not assigned there (Weng et al., 2011). It is important to note that in that work the lignin was acetylated and, therefore, the chemical shifts of the amentoflavone signals were slightly different (this point was confirmed by acetylating *S. kraussiana* and recording HSQC under the same experimental conditions). Therefore, the present work reports for the first time the presence of amentoflavone in the lignin fraction of a *Selaginella* species. The occurrence of amentoflavone explains the appearance of 4-hydroxyacetophenone (peak 22) in the pyrograms of the *S. kraussiana* lignin (Figure 2) since 4-hydroxyacetophenone was the main compound released by pyrolysis of an amentoflavone standard (data not shown).

#### Horsetails

Despite being a vascular plant, the lignin-like preparation from the horsetail *E. palustre* (class Polypodiopsida, order Equiseales) did not yield significant amounts of pyrolysis lignin markers and only traces of phenol and 4-methylphenol, which could arise from proteins and not from real H-lignin units, could be detected (Figure 2 peaks 1 and 4, respectively). This could be related to the limited development of its vascular system with no secondary thickening (Logan and Thomas, 1985) and its wetland-adapted lifestyle. The HSQC spectrum showed significant amounts of proteins, which would explain the release of phenol and 4-methylphenol upon Py-GC/MS and confirm the absence of H-lignin units (Supplementary Figure 2). Lignin-derived compounds have been previously reported, although in very low amounts, in other horsetails by chemical degradation of whole cell-wall material (Logan and Thomas, 1985; Espiñeira et al., 2011).

#### Ferns

The aromatic regions of the HSQC spectra (Figure 5) of the lignin-like preparations isolated from the ferns *P. aquilinum* and *N. cordifolia* (class Polypodiopsida, order Polypodiales) showed almost exclusively signals for G-lignin (G_2_, G_5_, and G_6_), together with small signals for H-lignin units in the spectrum of *P. aquilinum*. Correlation signals of coniferaldehyde end-groups (J_2_, J_6_, and J_8_) and flavonoids were also detected.

In the case of *P. aquilinum*, the flavonoid signals were unambiguously assigned to kaempferol (by comparison with an authentic standard, Supplementary Figure 1), whereas in the case of *N. cordifolia*, it was not possible to conclusively assign them (unknown signals labeled with question marks) (Figure 5). The occurrence of kaempferol in *P. aquilinum* is not limited to the lignin fraction but its presence, mainly as kaempferol glycosides, has also been reported in the ethanol extracts (Nakabayashi, 1955; Imperato, 1996).

Analysis of signals in the aliphatic-oxygenated region of the spectra showed that *P. aquilinum* and *N. cordifolia* present a rather similar linkages distribution (Table 3). Apart from the signal of methoxyl groups (in G-units), typical signals of different inter-unit linkages were clearly detected, including β−*O*−4′ alkyl-aryl ethers (A), β−5′ phenylcoumarans (B), β−β′ resinols (C) and 5−5′ dibenzodioxocins (D).

#### Ancient Gymnosperms (Cycads and Gnetophytes)

To have a broader view on lignin in early plants, the lignin-like fractions of two ancestral gymnosperms (division Spermatophyta) with secondary thickening, *C. revoluta* (class Cycadopsida) and *E. fragilis* (class Gnetopsida), were also studied in some detail by HSQC NMR (Figure 6).

The aromatic region of the HSQC spectrum of the *C. revoluta* lignin-like fraction was clearly dominated by correlations signals corresponding to G-lignin units. Cross-signals of H-lignin units, coniferaldehyde end-groups (J) and flavonoids were also detected, albeit in considerably lower amounts. The flavonoid signals were assigned to amentoflavone (Am) and naringenin (N) moieties with the aid of authentic standards (Supplementary Figure 1). The presence of amentoflavone in the lignin fraction is not surprising as several amentoflavone-derived compounds have been reported among the *C. revoluta* extractives (Moawad et al., 2010, 2014). In the aliphatic-oxygenated region of the spectrum, typical signals of different lignin substructures, including β−*O*−4′ alkyl-aryl ether (A), β−5′ phenylcoumaran (B), β−β′ resinol (C) and 5−5′ dibenzodioxocin (D) linkages were also detected. The relative abundances of the above substructures are provided in Table 3.

In contrast, the HSQC spectrum of the *E. fragilis* lignin revealed an H-G-S type polymer enriched in S-lignin units (S/G ratio of 2.6). Similar lignin composition in Gnetopsida species have been observed in the related *Ephedra viridis* Coville by thioacidolysis (Ros et al., 2007), *Ephedra sinica* Stapf by CuO alkaline degradation (Jin et al., 2007b) and *Gnetum gnemon* L. by ozonation and NMR (Nawawi et al., 2016). Therefore, the appearance of S-lignin in *E. fragilis* is another case of parallel and convergent evolution of S-lignin between primitive vascular plants and angiosperms. The HSQC analysis of the *E. fragilis* lignin also revealed structural details (linkages and substructures). In this way, we found that the S-enriched lignin of *E. fragilis* is mainly composed of β−*O*−4′ substructures (81%), followed by lower amounts of β−β′ resinols (11%) and β−5′ phenylcoumaran (7%) and the absence of dibenzodioxocins (Table 3). The lower contents of dibenzodioxocin and phenylcoumarans in the *E. fragilis* lignin-like fraction, compared with *C. revoluta*, seems to be related to its higher S/G ratio. Phenylcoumaran structures are composed of at least one G-lignin unit (and the second can be either G- or S-), while two G-lignin units are involved in the dibenzodioxocin structures (S-units cannot form dibenzodioxocin structures). In addition, signals from flavonoids were also detected in the aromatic region of the spectrum (Figure 6), some of which were assigned to the flavonol kaempferol by comparison with an authentic standard (Supplementary Figure 1). As found in the abovementioned *Selaginella*, investigated as a reference system for metabolic evolution (Weng and Noel, 2013), lignin in *E. fragilis* and some other Gnetales species have also been found to incorporate S-units (Logan and Thomas, 1985) through convergent evolution.

## Conclusion

Lignins from precursors synthesized through the classical monolignol biosynthetic route (yielding *p-*coumaryl, coniferyl and sinapyl alcohols) were not significantly detected in the two moss species, as the more ancestral plants analyzed. In agreement with their contribution to land colonization by vascular plants, G lignins were found from lycophytes to ferns and ancient gymnosperms (cycads and gnetophytes). However, they were hardly detected in the gnetophyte *E. palustre* characterized by its limited vascular development. Then, these G-type primitive lignins, which would also include flavonoids and minor H-type units, incorporated S-type units in several parallel and convergent evolutionary events. In this way, we found S-rich lignins in the lycophyte *S. kraussiana* (S/G ratio of 2.1) and the gnetophyte *E. fragilis* (S/G ratio of 2.6), before their final acquisition by modern angiosperms. In this way, the presence of S lignin paralleled trunk secondary thickening in different groups of plants characterized by their extant and/or ancestral arborescent development. In fact, it has been suggested that S-lignin units would have appeared up to five times during plant evolution (Novo-Uzal et al., 2012).

Plant flavonoids and lignin-like polymers have existed concomitantly since the first land plants appeared. They are believed to play a key role as UV screens due to their aromatic and partially phenolic nature (Clayton et al., 2018; Davies et al., 2020), although it is likely that the amount of flavonoids in the lignin-like fractions has been reduced as terrestrial plants have evolved. To a greater or lesser extent, flavonoids were detected within the lignin-like fractions of all the primitive plants studied here (from mosses to ancient gymnosperms). The B-ring flavonoids derives from the general phenylpropanoid biosynthetic pathway. Interestingly, all the flavonoids identified in the lignin-like preparations present a B-ring similar to the H-lignin units (as found in apigenin, naringenin and kaempferol). Flavonoids with a B-ring similar to G- and S-lignin units, and even in the form of catechol or pyrogallol, have been reported in extracts of most of these primitive plants (Nakabayashi, 1955; Geiger et al., 1997; Weng and Noel, 2013; Negm et al., 2016) but, due to unknown reasons, they do not incorporate into their lignin-like fractions.

Flavonoid incorporation into the lignin polymer is not a new topic. It was described that tricin [5,7-dihydroxy-2-(4-hydroxy-3,5-dimethoxyphenyl)-4H-chromen-4-one] is incorporated into the lignin of wheat straw and other monocots (del Río et al., 2012; Rencoret et al., 2013; Lan et al., 2015, 2016b). However, this is in contrast to ancestral species that do not incorporate G/S-type B-ring containing flavonoids, as mentioned above. Other flavonoids, such as apigenin and naringenin, are also compatible with lignification, as shown in genetically-modified plants (Lam et al., 2017, 2019). In the present work, we report for the first time the presence of naringenin, apigenin, kaempferol, and amentoflavone in the lignin-like fraction isolated from several wild ancestral plants, as confirmed by the corresponding HSQC signals in flavonoid-containing *in vitro* synthesized DHPs. Research is currently underway to confirm whether these flavonoids are covalently bound to the lignin polymer.

## Data Availability Statement

The raw data supporting the conclusions of this article will be made available by the authors, without undue reservation.

## Author Contributions

JR: work design and supervision, methodology development, chemical characterization of lignin, and contribution to first and final draft writing. AG: funding acquisition and final draft writing. GM: isolation of lignin-like fractions. JCdR: contribution to pyrolysis and NMR data interpretation. YT and PYL: DHP preparation. MP-B and FJR-D: sample preparation. JMB: collection of plant materials. ATM: work design, supervision, and contribution to first and final draft writing. All authors contributed to the article and approved the version submitted.

## Conflict of Interest

The authors declare that the research was conducted in the absence of any commercial or financial relationships that could be construed as a potential conflict of interest.

## Publisher’s Note

All claims expressed in this article are solely those of the authors and do not necessarily represent those of their affiliated organizations, or those of the publisher, the editors and the reviewers. Any product that may be evaluated in this article, or claim that may be made by its manufacturer, is not guaranteed or endorsed by the publisher.
