# Supplementary material for: New Insights on Structures Forming the Lignin-Like Fractions of Ancestral Plants
Source: Front Plant Sci. 2021 Oct 7;12:740923. doi: 10.3389/fpls.2021.740923 (PMC8528957; doi:10.3389/fpls.2021.740923)
Supplement: Supplementary file 1 [file Data_Sheet_1.docx]

SUPPLEMENTARY INFORMATION

New insights on structures forming the lignin-like fractions of ancestral plants

*Jorge Rencoret^1^*, Ana Gutiérrez^1^, Gisela Marques^1^, José C. del Rio^1^, Yuki Tobimatsu^2^, Pui Y. Lam^2^, Marta Pérez-Boada^3^, Francisco Javier Ruiz-Dueñas^3^, José M. Barrasa^4^, Angel T. Martínez^3^**

*^1^Instituto de Recursos Naturales y Agrobiología de Sevilla (IRNAS), CSIC, Seville, Spain*

*^2^Research Institute for Sustainable Humanosphere, Kyoto University, Kyoto, Japan*

*^3^Centro de Investigaciones Biológicas "Margarita Salas" (CIB), CSIC, Madrid, Spain*

*^4^Departamento de Biología Vegetal, Universidad de Alcalá, Alcalá de Henares, Spain*

**Correspondence: Jorge Rencoret (*[*jrencoret@irnase.csic.es*](mailto:jrencoret@irnase.csic.es)*), Angel T. Martínez (*[*atmartinez@cib.csic.es*](mailto:atmartinez@cib.csic.es)*)*

Supplementary information shows HSQC spectra of naringenin, apigenin, kaempferol and amentoflavone standards along with those of DHPs from coniferyl alcohol/naringenin and coniferyl alcohol/apigenin (**Fig. S1**); aliphatic-oxygenated and aromatic regions of the HSQC spectra of the *P. commune* and *E. palustre* "lignin-like" fractions (**Fig. S2**); **iii)** aromatic regions of the HSQC spectra of the dioxane-lignin and the MWL preparations isolated from *S. kraussiana* (**Fig. S3**).

**
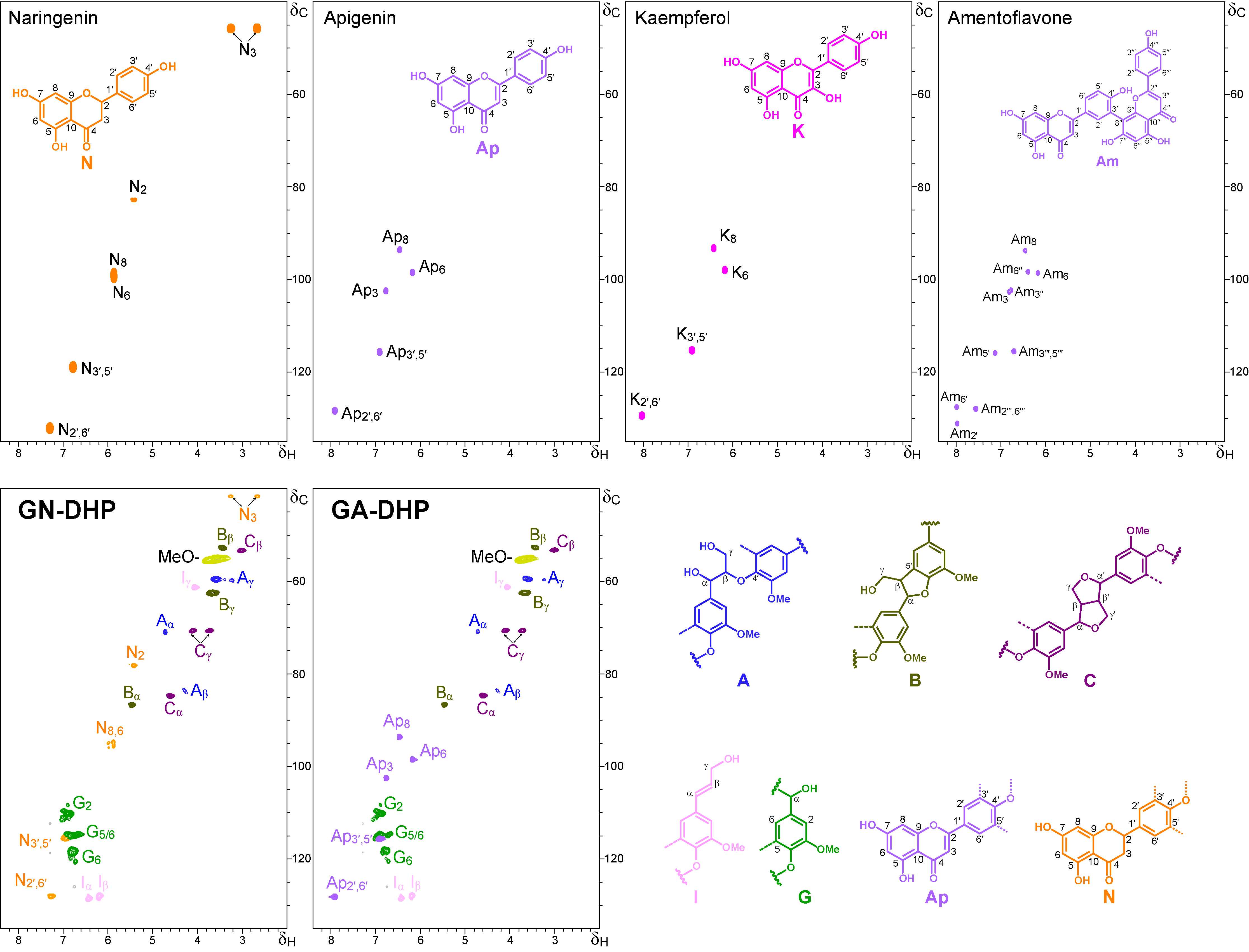
**

**FIGURE S1.** HSQC spectra of naringenin, apigenin, kaempferol and amentoflavone standards along with those of DHPs from coniferyl alcohol/naringenin (GN-DHP) and coniferyl alcohol/apigenin (GA-DHP). The main structures identified in the GN-DHP and GA-DHP spectra −i.e. β-*O*-4' ether (**A)**, β-5' phenylcoumaran (**B**) and β-β' resinol **(C)** linkages, coniferyl alcohol end groups **(I)**, guaiacyl (**G**) units, and apigenin (**Ap**) and naringenin (**N**) moieties− are also shown.


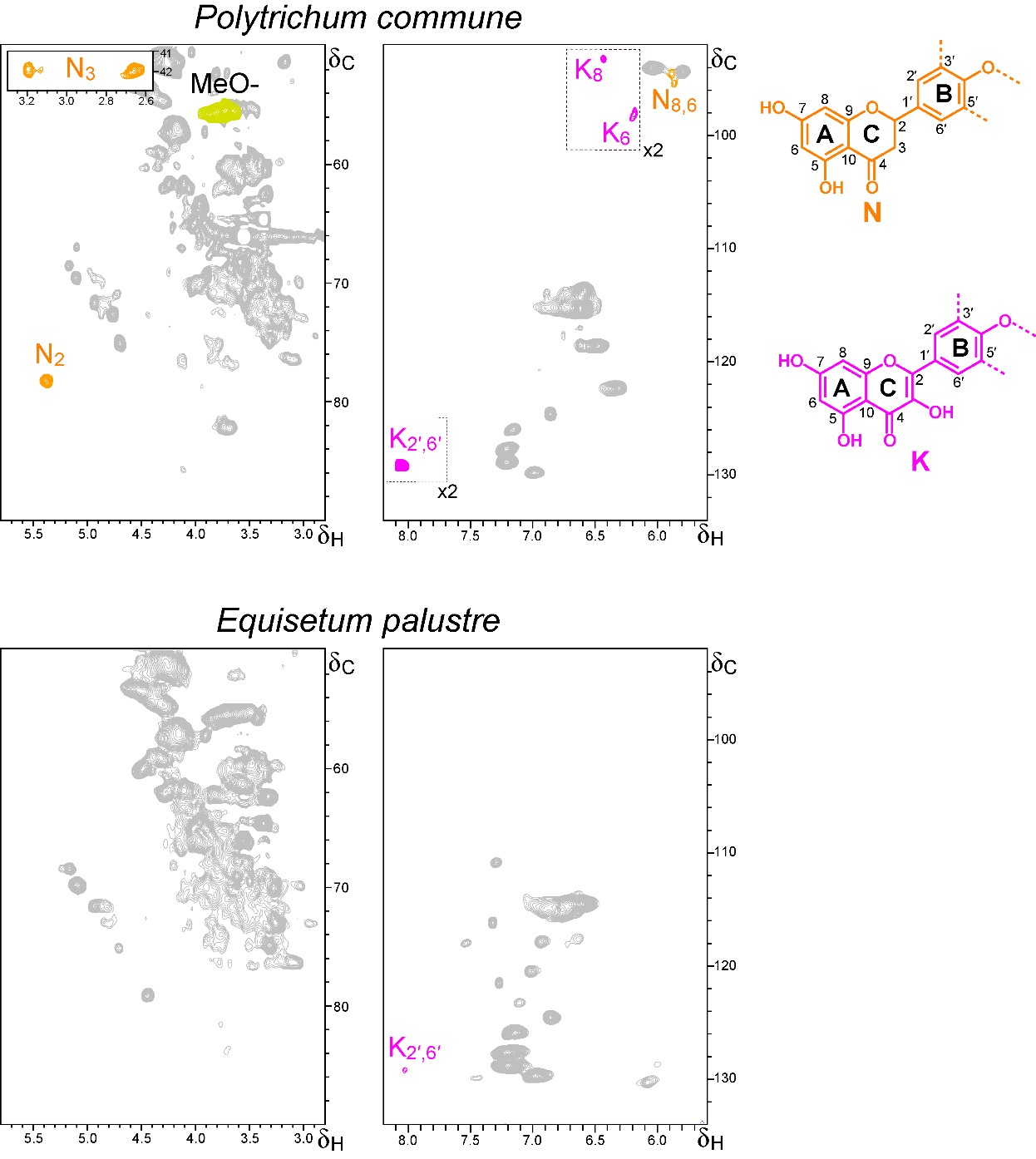


**FIGURE S2.** Aliphatic-oxygenated (δ_C_/δ_H_ 50–90/2.8–5.8) and aromatic (δ_C_/δ_H_ 92–134/5.5–8.2) regions of the HSQC spectra of the *P. commune* and *E. palustre "*lignin-like" fractions*.* Due to the low lignin content, the spectra are saturated by protein signals, and only minor signals of kaempferol (**K**) and naringenin (**N**) units can be observed.

**
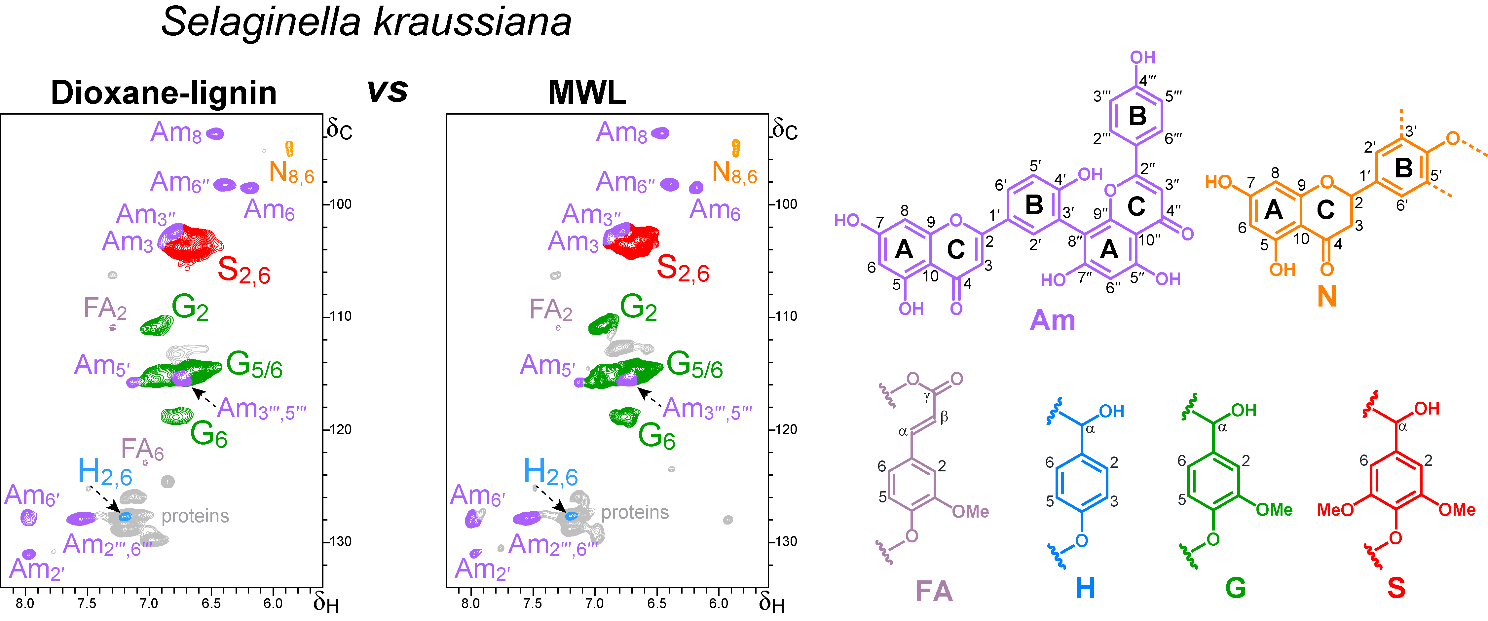
**

**FIGURE S3.** Aromatic regions of the HSQC spectra of the dioxane-lignin and the MWL preparations isolated from *S. kraussiana*. The main structures identified are depicted on the right.
